# Multiplexed Microfluidic Cartridge for At-Line Protein Monitoring in Mammalian Cell Culture Processes for Biopharmaceutical Production

**DOI:** 10.1021/acssensors.0c01884

**Published:** 2021-03-16

**Authors:** Inês F. Pinto, Ruben R. G. Soares, Meeri E.-L. Mäkinen, Veronique Chotteau, Aman Russom

**Affiliations:** † KTH Royal Institute of Technology, Division of Nanobiotechnology, Department of Protein Science, Science for Life Laboratory, 171 21 Solna, Sweden; ‡ KTH Royal Institute of Technology, Department of Industrial Biotechnology, School of Engineering Sciences in Chemistry, Biotechnology and Health, 106 91 Stockholm, Sweden; § AdBIOPRO, Competence Centre for Advanced BioProduction by Continuous Processing, KTH, 100 44 Stockholm, Sweden; ∥ AIMES, Center for the Advancement of Integrated Medical and Engineering Sciences at Karolinska Institutet and KTH Royal Institute of Technology, 100 44 Stockholm, Sweden

**Keywords:** microfluidics, streptavidin beads, immunoassay, colorimetric, monoclonal antibodies, host cell proteins

## Abstract

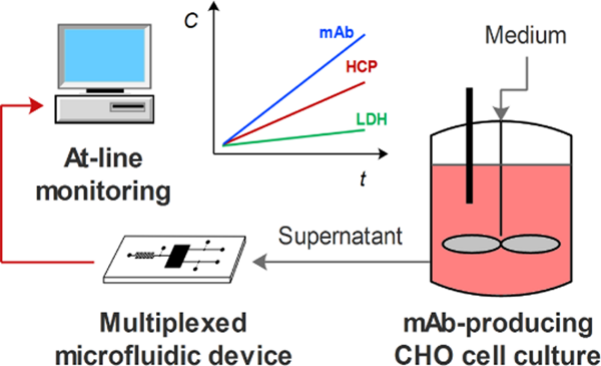

The biopharmaceutical market has been rapidly growing in recent years, creating a highly competitive arena where R&D is critical to strike a balance between clinical safety and profitability. Toward process optimization, the recent development and adoption of new process analytical technologies (PAT) highlight the dynamic complexity of mammalian/human cell culture processes, as well as the importance of fine-tuning and modeling key metabolites and proteins. In this context, simple, rapid, and cost-effective devices allowing routine at-line monitoring of specific proteins during process development and production are currently lacking. Here, we report the development of a versatile microfluidic protein analysis cartridge allowing the multiplexed bead-based immunodetection of specific proteins directly from complex mixtures with minimal hands-on time. Colorimetric quantification of Chinese hamster ovary (CHO) host cell proteins as key impurities, monoclonal antibodies as target biopharmaceuticals, and lactate dehydrogenase as a marker of cell viability was achieved with limits of detection in the 1–10 ng/mL range and analysis times as short as 30 min. The device was further demonstrated for the monitoring of a Rituximab-producing CHO cell bioreactor over the course of 8 days, providing comparable recoveries to standard enzyme-linked immunosorbent assay (ELISA) kits. The high sensitivity combined with robustness to matrix interference highlights the potential of the device to perform at-line measurements spanning from the bioreactor to the downstream processing.

The global biopharmaceutical market has been continuously and rapidly expanding in the last 5 years with a compound annual rate of 10%,^1^ where 8 among the top 10 selling drugs at a global scale are biologics.^2^ In such a competitive market, biopharma companies are expected to increase R&D investment by 2.8% each year reaching $182 billion by 2022.^1^ The fraction of investment directed to process development focuses on improving manufacturing efficiency and flexibility^1^ from both upstream (cell culture) and downstream (purification and formulation) perspectives. Among all biopharmaceuticals, monoclonal antibodies (mAbs) are currently the dominant type of molecule with a ∼33% market share^1,3^ and four new mAbs being approved each year.^3^ Considering the high efficacy of these molecules in a range of chronic diseases, process intensification and streamlining to maximize capacity while achieving efficient, sustainable, and less expensive production^3^ is necessary to minimize the time to market and tackle the increasing pipelines of new therapeutics.^4^

On the upstream side, the manufacturing of glycoproteins relies mostly on Chinese hamster ovary (CHO) cells, since they have been demonstrated safe for more than three decades, provide very high productivities, deliver a glycosylation profile adequate for human therapy, and are very robust in large scale suspension culture using serum-free media.^5^ The production in bioreactor is followed by the downstream processing, which usually comprises cell removal, affinity chromatography, *e.g*., protein A for mAbs, cation/anion exchange polishing, viral inactivation, and a sequence of filtration steps.^6^ Although the field has made tremendous progress since the eighties, intense efforts are still aimed at productivity maximization^7,8^ and tuning of the protein quality. The success of these efforts is highly dependent on efficient and fit-for-purpose process analytical technology (PAT),^9^ which enables new knowledge for the monitoring, control, optimization, and modeling of the unit operations.^7^

A complete understanding of the cell culture process encompasses more than the monitoring of the few main parameters usually measured,^10,11^ namely, cell density and concentrations of glucose, glutamine, lactate, and ammonia, considering the hundreds of metabolites and proteins that are taken up or produced by the cells and dynamically change over time, both at supernatant and cell levels.^12,13^ The measurement of small molecules, including amino acids and sugars, is usually performed using liquid chromatography, capillary electrophoresis, or high-performance liquid chromatography-mass spectrometry (HPLC-MS), while high-molecular-weight proteins, including product titer and host cell proteins (HCP), are measured using affinity, *e.g*., enzyme-linked immunosorbent assay (ELISA),^14^ or mass, *e.g*., HPLC-MS.^15^ These techniques, typically performed off-line, can also be converted into at-line measurements when coupled with automatic sampling devices.^7^ On the downstream end, the analytics focus mostly on IgG quantification against other protein impurities, *e.g*., HCP, and are typically performed using automated liquid handling stations coupled with well-plate ELISAs or, alternatively, other recent affinity-based platforms coupled to fluorescence (Gyrolab or Ella)^16,17^ or optical interference effects (Octet system)^18^ for target detection. While these strategies provide semiautomated protein quantification, they rely on complex and expensive equipment combined with a total analysis time ranging from 1 to 5 h.^17,18^ Other recently reported microfluidic immunoassays for protein detection in the context of biomarker detection^19−21^ are also characterized by long analysis times (>40 min) with more than three individual steps and complex fabrication (*i.e*., dense microchannel footprints and/or cleanroom microfabrication), operation (*i.e*., internal valving), and signal transduction schemes (*i.e*., requiring single-particle imaging and/or fluorescence detection). Overall, PAT with high complexity and costs on both upstream and downstream ends hinders not only the routine monitoring of manufacturing but also the adaptation of quality by design (QbD) during development, due to equally high costs of design of experiment (DoE) approaches.^22,23^

To address the current gap in PAT and provide a general, simple, and cost-effective at-line quantification of proteins from cell culture to purified product, we developed and optimized a versatile microfluidic protein analysis cartridge allowing the multiplexed quantification of proteins directly from complex mixtures with minimal hands-on time. The device comprises four sub-microliter columns packed with agarose beads conjugated with specific antibodies/antigens to quantify three different proteins and one column serving as internal positive control. The generated colorimetric signal at bead level can be visually interpreted and quantified by white light transmission/scattering-based imaging. As a model system, we selected IgG (target product), CHO HCP (main impurities), and lactate dehydrogenase (LDH) (marker of cell viability) as key proteins, thus encompassing three key attributes of cultivation processes.

## Experimental Methods

### Modification of Agarose Beads

*N*-Hydroxysuccinimide (NHS)-activated Sepharose 4 Fast Flow (GE Healthcare) beads (mean particle size, 90 μm) were coupled to (i) streptavidin and (ii) LDH *via* primary amine groups in the proteins. For each modification protocol, a volume of 150 μL of bead slurry was washed with 1.5 mL of a cold 1 mM HCl solution.

#### Streptavidin-Coated Agarose Beads

Purified recombinant streptavidin (Thermo Fisher Scientific) was prepared in a coupling buffer (0.2 M NaHCO_3_, 0.5 M NaCl, pH 8.3) at a concentration of 1 mg/mL (*V* = 75 μL). The streptavidin solution was added to the washed preactivated beads at a ratio of 1:2 (v/v) and incubated at room temperature for 2 h with orbital agitation. The nonreacted groups on the beads were blocked by incubation with 1.5 mL of 0.1 M Tris–HCl, pH 8.5, for 1–2 h at room temperature with orbital agitation, followed by incubation with an additional 1.5 mL for 24 h at 4 °C. The blocking solution was then replaced with 1.5 mL of phosphate-buffered saline (PBS) pH 7.4 containing 0.02% sodium azide for long-term storage at 4 °C.

#### LDH-Coated Agarose Beads

l-Lactate dehydrogenase from rabbit muscle (Merck) was washed in an Amicon Ultra-0.5 centrifugal filter unit (Merck) with a cutoff of 10 kDa, to remove the high concentration of ammonium sulfate present in the stock formulation and maximize the coupling efficiency. Buffer exchange to an amine-free coupling buffer (see the previous section) was accomplished by spinning at 14 000*g* for 5 min. The process was repeated 3×, and the final concentration of LDH was adjusted to 1 mg/mL (*V* = 75 μL). The LDH solution was added to the washed preactivated beads at a ratio of 1:2 (v/v), and the following incubation and washing steps were as described in the previous section.

### Immunoassays and Antibody Functionalization

Modified agarose beads were used as solid phase to perform (i) sandwich-based or (ii) competitive-based immunoassays coupled with colorimetric detection of the target proteins. The preparation of biotinylated capture antibodies and horseradish peroxidase (HRP)-labeled detection antibodies was made in-house, using EZ-LinkNHS-Biotin (Thermo Fisher Scientific) and HRP conjugation kits (Abcam), respectively.

#### CHO HCP Sandwich Immunoassay on Streptavidin-Coated Beads

Affinity-purified goat anti-CHO HCP (Cygnus Technologies, 3G-0016-AF) was buffer-exchanged and concentrated to 5 mg/mL in PBS, using an Amicon Ultra-0.5 centrifugal filter unit with a cutoff of 100 kDa. Biotin was prepared in dimethyl sulfoxide (DMSO) at a concentration of ∼3.4 mg/mL and added to the concentrated antibody solution at a ratio of 1:19 (v/v). The mixture was incubated in the dark for 30 min with continuous agitation. Unreacted biotin was removed using an Amicon Ultra-0.5 centrifugal filter unit with a cutoff of 10 kDa, over five washing steps with PBS. The goat anti-CHO HCP antibody was also conjugated with HRP, according to the instructions provided by the supplier. CHO HCP target antigen was acquired as a concentrate solution (27 mg/L) from Cygnus Technologies (product code: F553H).

#### IgG Sandwich Immunoassay on Streptavidin-Coated Beads

Goat anti-human IgG Fc preadsorbed antibody (Abcam, ab98616) was conjugated with biotin, according to the protocol described in the previous section. Goat anti-human IgG H&L preadsorbed antibody (Abcam, ab7148) was conjugated with HRP. Native Human IgG protein (Abcam, ab98981) was used as the target antigen.

#### LDH Competitive Immunoassay on LDH-Coated Beads

Anti-lactate dehydrogenase antibody (Abcam, ab191332) was conjugated with HRP, and l-Lactate dehydrogenase from rabbit muscle (Merck) was used as the target antigen.

#### Internal Positive Control

Donkey anti-goat IgG H&L preadsorbed antibody (Abcam, ab7120) was conjugated with biotin, according to the protocol previously described. The conjugated antibody was then bound to streptavidin-coated agarose beads to target all HRP-labeled antibodies, which are goat polyclonal antibodies against each target antigen.

### Fabrication of Microfluidic Devices

The bead-based experiments were performed in single-plexed or multiplexed microfluidic devices fabricated in poly(dimethylsiloxane) (PDMS) using standard mold replication techniques as reported in detail elsewhere,^24^ apart from minor differences described herein. The devices were designed using AutoCAD (Autodesk, education license). Aluminum hard masks and SU-8 mold were purchased from INESC Microsystems and Nanotechnologies (INESC-MN, Lisbon, Portugal). Photos and schematics with details of the fabrication procedure and channel dimensions of the devices are shown in the Supporting Information (Figure S1). The PDMS was prepared by adding the curing agent to the prepolymer in a ratio of 1:10 (w/w) and baked for 2 h in a convection oven at 65 °C. The devices were then peeled off of the mold, and access holes for inlets and outlets were punched using blunt syringe needles. The devices were sealed against plain glass slides (Corning) following exposure to an oxygen plasma treatment for 30 s (Femto Science CUTE, 100 W, 600 mTorr O_2_).

### Device Operation and Liquid Handling

Prior to introduction in the microfluidic device, the streptavidin-coated agarose beads were conjugated with the corresponding biotinylated antibodies. A volume of 4 μL of bead slurry was added to 20 μL of antibody solution and incubated for 30 min with agitation. In the case of LDH-coated beads, which were used in a competitive assay, 4 μL of bead slurry was incubated with 20 μL of PBS, to simulate the multiplexing conditions. After incubation, the beads were washed with PBS to remove nonconjugated antibodies and suspended in a solution of 20% (w/w) poly(ethylene glycol) (PEG) 8000.

**Figure 1:**
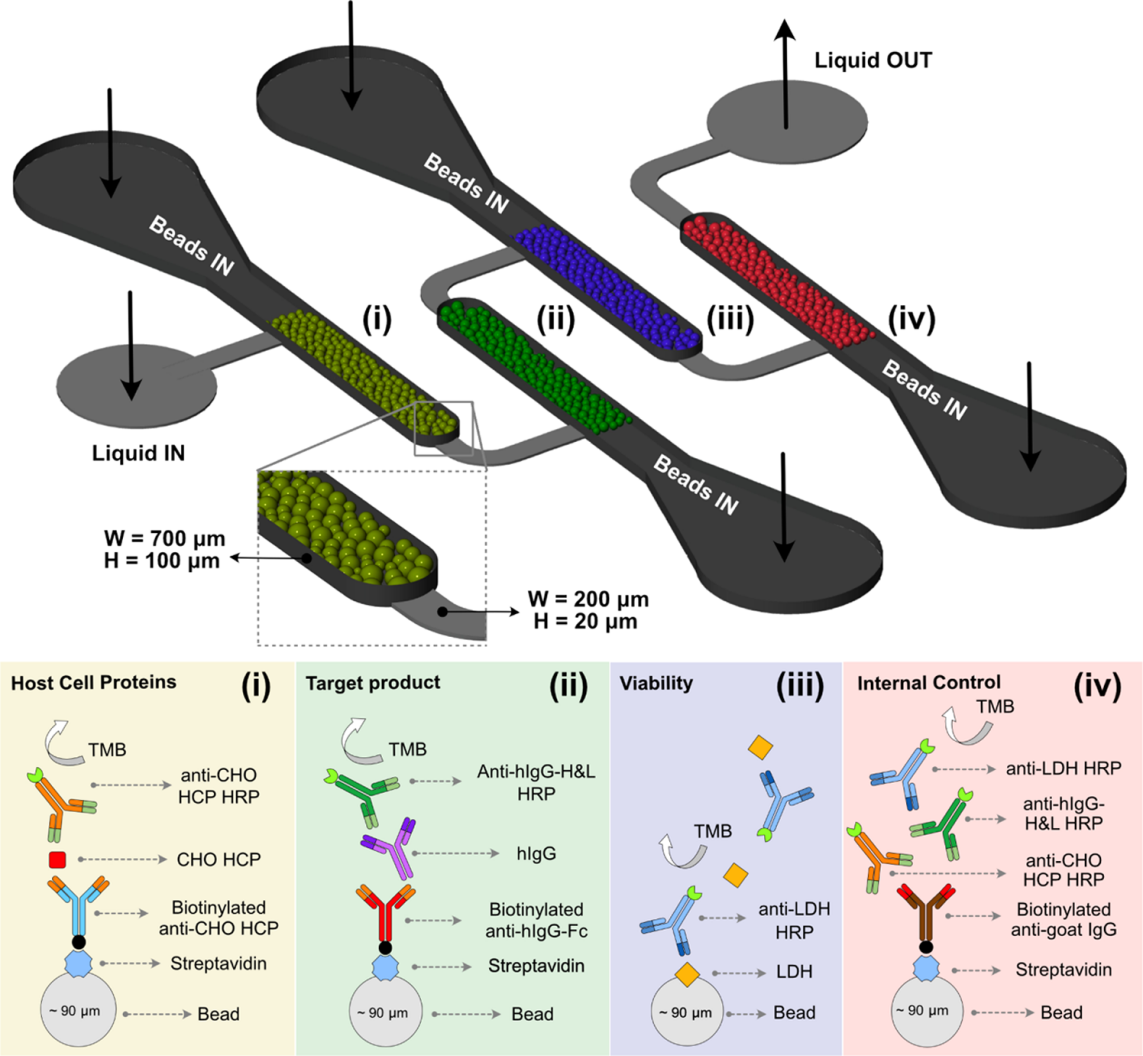
Schematics of the microfluidic cartridge used for bead-based immunoassays. The multiplexed device comprises four interconnected columns filled with differently conjugated beads, each targeting a specific protein: (i) CHO HCP, (ii) IgG, (iii) LDH, and includes (iv) an internal positive control.

In the single-plexed devices, which consist of an array of individual straight columns, the beads were packed through a single inlet using a pipette tip as reservoir and liquid flow was driven by application of a negative pressure at the outlet using a syringe pump (NE-1200, New Era Pump System, Inc.). On the other hand, in the multiplexed devices, the beads were manually and sequentially introduced in the columns using a pipette in dedicated inlets, which were then sealed with a metal plug to allow a subsequent liquid flow transversal to the four bead-packed columns using a positive pressure at the inlet (Figure 1). In both devices, the beads (average particle size ranging from 45 to 165 μm) were trapped at the interface between two microchannels with heights of 100 and 20 μm. Details regarding the bead packing procedure for the multiplexed devices are shown in the Supporting Information (Table S1).

Following bead packing, the channels were washed with PBS at 5.7 μL/min for 3 min and blocked with 1% (w/v) casein solution (Thermo Fisher Scientific) at 2.8 μL/min for 10 min. The samples were continuously applied at 1.7 μL/min for 30 min, and the channels were subsequently washed twice with 1× PBS, 0.05% Tween at 5.7 μL/min for 2 min. Signal development on the beads was performed by flowing a 3,3’,5,5’-tetramethylbenzidine (TMB)-blotting substrate solution (Thermo Fisher Scientific) at 5.7 μL/min for 5 min. The assay steps were the same in both devices; however, the flow rates were adjusted to ensure the same liquid velocity through the beads, considering the different cross-sectional areas of the single-plexed (0.07 mm^2^) and multiplexed (0.04 mm^2^) devices. For simplicity, the flow rates indicated herein correspond only to those used in the multiplexed device. All devices were used for a single assay and subsequently discarded.

The samples containing the target proteins were prepared in a diluent buffer (Cygnus Technologies, product code I028), unless stated otherwise.

### Signal Acquisition and Image Processing

The bead-packed channels were imaged using a flatbed scanner (Epson Perfection V800 Photo) with a resolution of 2400 dpi, and the colorimetric signal was measured using ImageJ (NIH) by averaging the area with beads relative to the background channel.

### CHO Cell Culture and Protein Quantification Using Standard Methods

Samples were collected from days 1, 3, 5, 7, and 8 of a fed-batch cultivation carried out in a 4 L benchtop bioreactor (Belach Bioteknik, Stockholm, Sweden). The cells were Rituximab-producing Chinese hamster ovary cells (TurboCellTM, kindly provided by Rentschler Biopharma, Laupheim, Germany). The base and feed media were chemically defined proprietary media without animal-derived components. CHO HCP concentrations were quantified using a CHO HCP ELISA kit, 3G (F550) from Cygnus Technologies. Rituximab concentrations were quantified using an IgG (total) Human ELISA kit (BMS2091) from Thermo Fisher Scientific and purified Rituximab as internal standard. For both ELISA kits, all samples and calibration wells were measured in duplicate according to the instructions from the suppliers. LDH concentrations were determined with triplicate measurements from centrifuged (180*g* for 5 min) samples with Cedex Bio Analyzer (Roche Diagnostics Deutschland GmbH, Mannheim, Germany).

## Results and Discussion

### Microfluidic Cartridge for Monitoring of CHO HCP, IgG, and LDH

The schematic of the microfluidic cartridge for bead-based immunoassays is shown in Figure 1. It comprises four bead columns, interconnected by a shallower channel that prevents the cross-talk between different types of beads and allows a homogeneous liquid flow through all of the columns, irrespective of variations in the bead density in each column.

The operation of the multiplexed cartridge assumes that the device is prefilled with beads conjugated to corresponding capture antibodies (sandwich immunoassay, Figure 1i,ii) or antigen (competitive immunoassay, Figure 1iii) and the sample under analysis is preincubated with all HRP-labeled detector antibodies at optimized concentrations. At-line monitoring and quantification of key proteins, namely, CHO HCP, IgG, and LDH, throughout the cell cultivation process and subsequent downstream processing is accomplished by flowing the sample combined with detector antibodies through the beads at approximately 1 column volume per second for a certain time. The continuous flow of solution minimizes mass-transport limitations along the column and reduces time to achieve equilibrium conditions.^25,26^ After flowing the sample, a colorimetric signal is generated using a TMB-blotting substrate solution, continuously flowed through the device. The enzymatic reaction with TMB results in the formation of blue precipitates that continuously accumulate on the beads and are not washed away by the liquid flow, contrarily to soluble TMB substrate typically used in ELISAs, which would be suboptimal in this case. The colorimetric signal is directly (sandwich) or inversely (competitive) proportional to the concentration of target protein in the sample. In this agarose bead-based setup, this colorimetric signal was previously observed to be at least 10-fold more sensitive than organic fluorophores coupled with fluorescence measurements,^26^ due to the possibility of accumulating signal over time. After developing the signal, the device is discarded since regeneration protocols to efficiently remove the captured antigens and wash the colored precipitates may hinder the performance of the biotinylated anti-IgG and anti-CHO HCP antibodies. In the particular case of LDH, a competitive assay design was selected since several combinations of pairs of commercial anti-rabbit and anti-human LDH antibodies using the respective antigen (rabbit LDH, Merck or human LDHA protein, ab93699, Abcam), namely, NBP1-48336B (polyclonal, Novus Biologicals), MABC150 (monoclonal, clone 5D 2.1, Merck), CSB-PA00045B0Rb-100 (polyclonal, Cusabio), and AF14A11 (monoclonal, Thermo Fisher), as well as the antibody used in the reported competitive assay, failed to provide a positive signal response in a sandwich configuration at LDH concentrations of up to 1 μg/mL. This limitation is hypothesized to be due to the high conservancy of LDH among mammals,^27^ thus failing to elicit an immune response against more than one epitope.

The last bead column includes an internal positive control (Figure 1iv), comprising streptavidin-coated beads conjugated to a biotinylated anti-goat antibody, which captures the three HRP-labeled detector antibodies spiked in the sample, irrespective of the presence or concentration of the protein targets. Thus, a colorimetric signal should be observed in this column in all cases, and the absence of this signal might indicate (i) possible issues in the sample flow through the fluidics or obstructions in the interface region of different columns, and (ii) possible lack of function of the capture antibody, assumed also for antibodies on adjacent bead columns. At the specific flow rate conditions used for the assay, the order in which the columns are packed relative to the inlet was observed to have a negligible impact on analyte capture and signal generation (Figure S2). This implies that the linear velocity and assay time were sufficient to avoid mass-transport limitations and depletion along the columns.

### Optimization of Immunoassay Parameters

**Figure 2:**
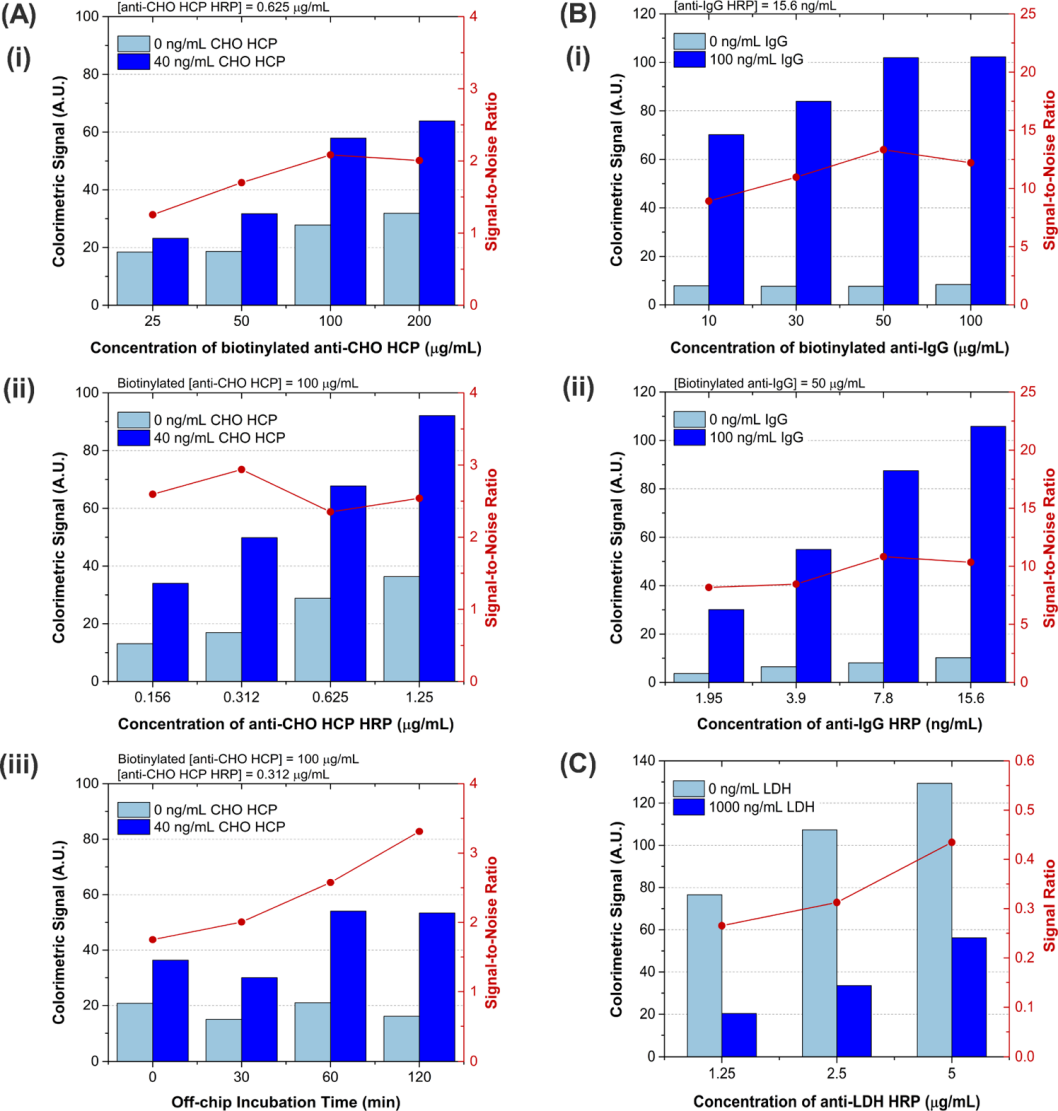
Optimization of immunoassay parameters for (A) CHO HCP, (B) IgG, and (C) LDH detection. Evaluated conditions included varying the (i) concentration of biotinylated capture antibody incubated with the beads, (ii) concentration of HRP-labeled detector antibody, and (iii) off-chip incubation time of target and detector antibody.

Each immunoassay was individually optimized in single-plexed microfluidic bead channels in terms of (i) concentration of biotinylated capture antibody incubated with the beads, (ii) concentration of HRP-labeled detector antibody incubated with the target antigen, and (iii) off-chip incubation time of detector with antigen. The colorimetric signal at a given concentration of target molecule was evaluated under the different conditions against a blank sample, and signal-to-noise ratios were calculated (Figure 2).

Considering the optimization of the capture antibody on streptavidin-coated beads, increasing concentrations of biotinylated anti-target showed a steady increase in the signal-to-noise ratio, both for CHO HCP (Figure 2Ai) and IgG (Figure 2Bi), until a plateau was reached. It is clear that increasing the concentration of capture antibody beyond a certain threshold is not beneficial for the assay, as steric hindrance effects on the surface and pores of the beads prevent the target molecules from being efficiently captured. Thus, concentrations of biotinylated anti-CHO HCP and anti-IgG of 100 and 50 μg/mL, respectively, were selected to subsequently evaluate the concentration of HRP-labeled detector antibody in the corresponding assays.

The effect of increasing concentrations of detector antibody was overall not very significant, as it resulted in an increase in both the specific and nonspecific (0 ng/mL target) signals, which translated into approximately constant signal-to-noise ratios. As the selection criteria were to maximize the signal-to-noise ratio for each parameter under analysis, concentrations of anti-CHO HCP HRP (Figure 2Aii) and anti-IgG HRP (Figure 2Bii) of 0.312 and 7.8 ng/mL, respectively, were selected.

The effect of preincubating the target molecule with the detector antibody prior to flowing the solution through the packed beads was also evaluated for the CHO HCP (Figure 2Aiii). The results show that, although it is still possible to differentiate between 0 and 40 ng/mL CHO HCP without a preincubation step, the signal-to-noise ratio is significantly maximized when the molecules are allowed to incubate for 2 h. Thus, an off-chip incubation step of 2 h was established for all immunoassays.

In the case of LDH, the assay was based on the competitive effect between LDH immobilized on the beads and LDH in solution, which means that the maximum signal is achieved in conditions were there is no target in the sample (0 ng/mL LDH). The decrease in signal in the presence of 1000 ng/mL LDH was evaluated at different concentrations of HRP-labeled detector antibody (Figure 2C), and the lowest signal ratio, *i.e*., highest sensitivity, was obtained at 1.25 μg/mL anti-LDH HRP.

### Concentration Curves and Immunoassay Performance

**Figure 3:**
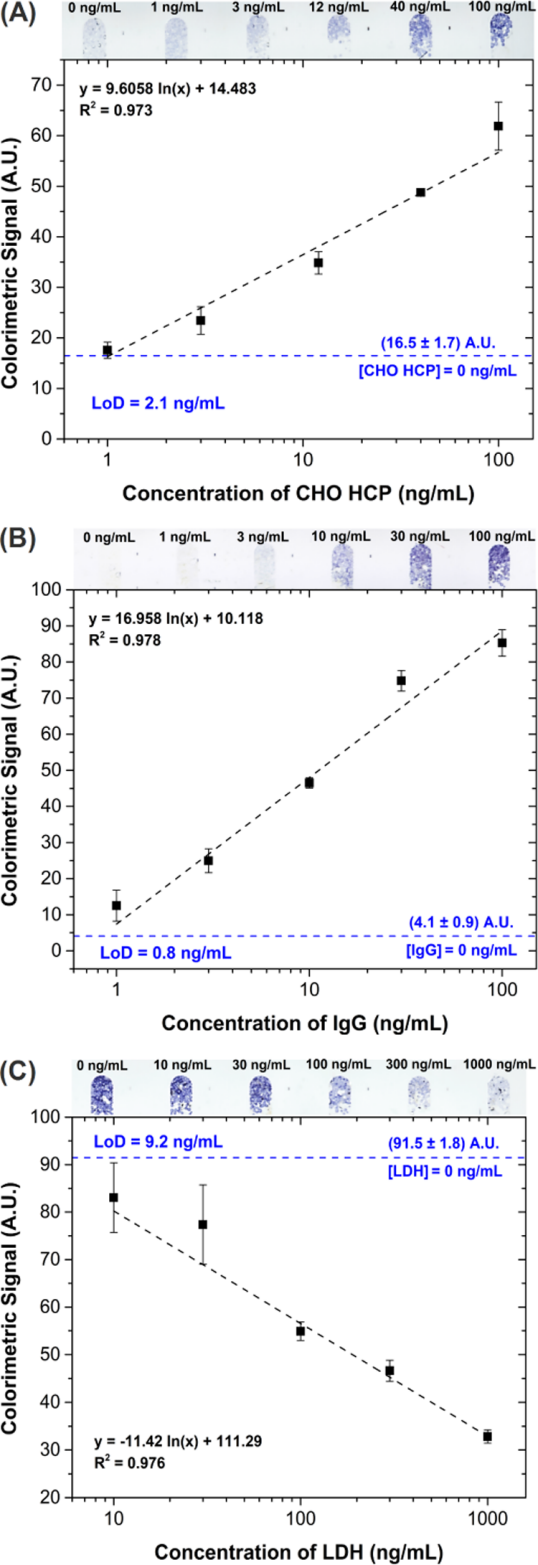
Concentration curves obtained for (A) CHO HCP, (B) IgG, and (C) LDH. Error bars correspond to the standard deviation of three independent measurements of each concentration. The horizontal dashed lines indicate the signal of the negative control (0 ng/mL of target), and limits of detection were determined considering 3.29σ of the negative control. Images of the bead-packed single microchannels were acquired with a flatbed scanner and are shown above each curve.

The optimal immunoassay parameters discussed in the previous section were then used to obtain calibration curves for each protein target (Figure 3). A linear response of the colorimetric signal was measured over 2 orders of magnitude of target concentration, and limits of detection of 2.1, 0.8, and 9.2 ng/mL were calculated for CHO HCP, IgG, and LDH, respectively.

Considering the high sensitivity and broad dynamic range of the assays, their application to monitor both the upstream and downstream processing of biopharmaceuticals can be envisioned. For the upstream monitoring, sample dilution is likely to be required in late stages of the cultivation process, as the concentration of these targets in a bioreactor can reach concentrations in the mg/mL range. On the other hand, quantification of impurity levels on the downstream side can be made directly from the undiluted sample, while meeting the regulatory limits of CHO HCP required for human administration, typically within a range of 1–100 ng/mL, and achieving a detection limit comparable with state-of-the-art immunodetection technologies.^18^

### Multiplexed Detection of Protein Targets

**Figure 4:**
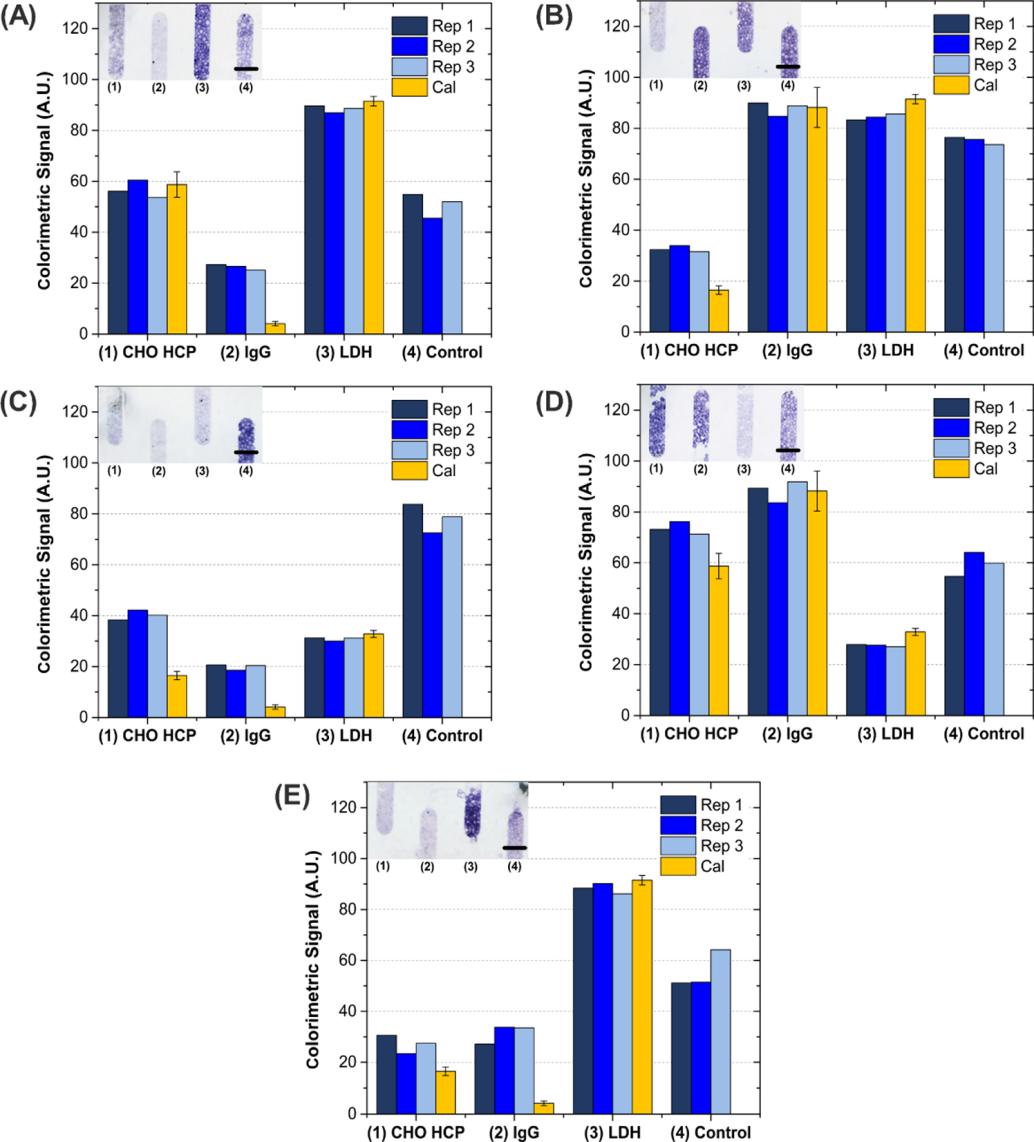
Multiplexed analysis of the protein targets and comparison with corresponding signals obtained in a single-plexed format (Cal). Artificial mixtures of the targets at different concentrations were as follows: (A) [CHO] = 100 ng/mL, [IgG] = 0 ng/mL, [LDH] = 0 ng/mL; (B) [CHO] = 0 ng/mL, [IgG] = 100 ng/mL, [LDH] = 0 ng/mL; (C) [CHO] = 0 ng/mL, [IgG] = 0 ng/mL, [LDH] = 1000 ng/mL; (D) [CHO] = 100 ng/mL, [IgG] = 100 ng/mL, [LDH] = 1000 ng/mL; (E) [CHO] = 0 ng/mL, [IgG] = 0 ng/mL, [LDH] = 0 ng/mL. For each combination of targets in solution, experiments were carried out in triplicate (Rep 1, Rep 2, Rep 3). Images of bead-packed microchannels in the multiplexed device were acquired with a flatbed scanner and are shown as insets in each graph. Scale bar = 500 μm.

After evaluating the performance and dynamic range of each assay in a single-plexed format, the assays were tested in the multiplexed configuration according to Figure 1. To characterize the multiplexed immunoassays concerning dynamic range and potential cross-reactivity, combinations of the three targets, namely, CHO HCP, IgG, and LDH at (A) 100/0/0 ng/mL; (B) 0/100/0 ng/mL; (C) 0/0/1000 ng/mL; (D) 100/100/1000 ng/mL; and (E) 0/0/0 ng/mL, respectively, were tested, and the results are compiled in Figure 4. The results from three independent measurements using the multiplexed configuration were compared with the expected signal from the calibration curves. Overall, three key conclusions can be derived from the results. First, the baseline colorimetric signal of all assays (*i.e*., signal for 0 ng/mL of each target) increased to 20–30 A.U., which is a consequence of the higher concentration of HRP-labeled detector antibodies (∼1.57 μg/mL), thus implying a cumulatively higher nonspecific signal background. The increase in background signal results in an increase of the calculated limits of detection of CHO HCP and IgG to ∼11 ng/mL (5-fold increase) and ∼7 ng/mL (8-fold increase), respectively, nonetheless still within the requirements for both downstream and upstream applications. Second, an expected and required cross-talk between the CHO HCP and the LDH assay can be observed since, on the one hand, LDH is a highly conserved molecule among mammals and is part of the HCP panel used to raise immunity during generation of anti-HCP antibodies and, on the other hand, the pool of HCP spiked in the sample contains also LDH. This cross-talk can be observed for the CHO HCP assays comparing conditions where there is presence (Figure 4C,D) and absence (Figure 4A,B,E) of LDH in the sample. In the absence of LDH (Figure 4A), the positive signal for the CHO HCP assay is in agreement with the expected from the calibration curve, while the negative (Figure 4B) agrees with the shifted background observed for all assays. However, in the presence of LDH, both the negative (Figure 4C) and positive (Figure 4D) CHO HCP samples have a positive ∼13 A.U. shift in colorimetric signal. The cross-talk could be equally observed for the positive LDH assays in the presence of CHO HCP (Figure 4D), where the LDH signal was lower than that expected from the calibration curve, while an agreement with the calibration was observed in the absence of CHO HCP (Figure 4C). Third, the signal from the internal control was observed to have some variability, which is hypothesized to be a result of the low concentration of capture antibody (2.5 μg/mL) used to modify the streptavidin beads. Considering that the anti-goat capture antibody in this column binds to all three HRP-labeled detection antibodies in solution, a low antibody concentration had to be used on the beads, to obtain a signal intensity comparable to adjacent columns and prevent signal saturation. However, such a low concentration can also have a detrimental effect, since any residual degradation in the capture antibody quality over time will have a high impact in signal variability. Thus, coating the beads with a higher concentration of capture antibody would allow us to overcome this variability, but for that, the sensitivity of the assay would have to be reduced by selecting a capture antibody with a lower association constant (*K*_a_) so that the resulting signal could be maintained within the desired range.^28^ Nevertheless, for the intended purpose of the internal control in providing a negative/positive response to validate the functionality of the assay, this variability is well tolerated.

### Quantification of Protein Targets in CHO Cell Culture Supernatants

The output of each assay was first validated in the presence of undiluted cell culture medium (FMX-8), 10× diluted medium in Cygnus dilution buffer, and plain dilution buffer, and no significant differences were observed for each sample matrix (Figure S3). These results support the compatibility of the device with direct measurements of complex mixtures in the beginning of the bioreactor operation, as well as progressive serial dilutions at later stages of the process.

Before testing the device for the monitoring of a CHO cell bioreactor run, IgG assay conditions were adjusted for the detection of chimeric (Cetuximab and Rituximab) and humanized (Trastuzumab) monoclonal antibodies. Upon modifying the concentration of capture antibody to 5 μg/mL and detector antibody to 500 ng/mL, comparable sensitivity (LoD ∼1.3 ng/mL) and dynamic range (1–100 ng/mL) to those obtained for the model polyclonal human IgG were achieved (Figure S4).

**Figure 5:**
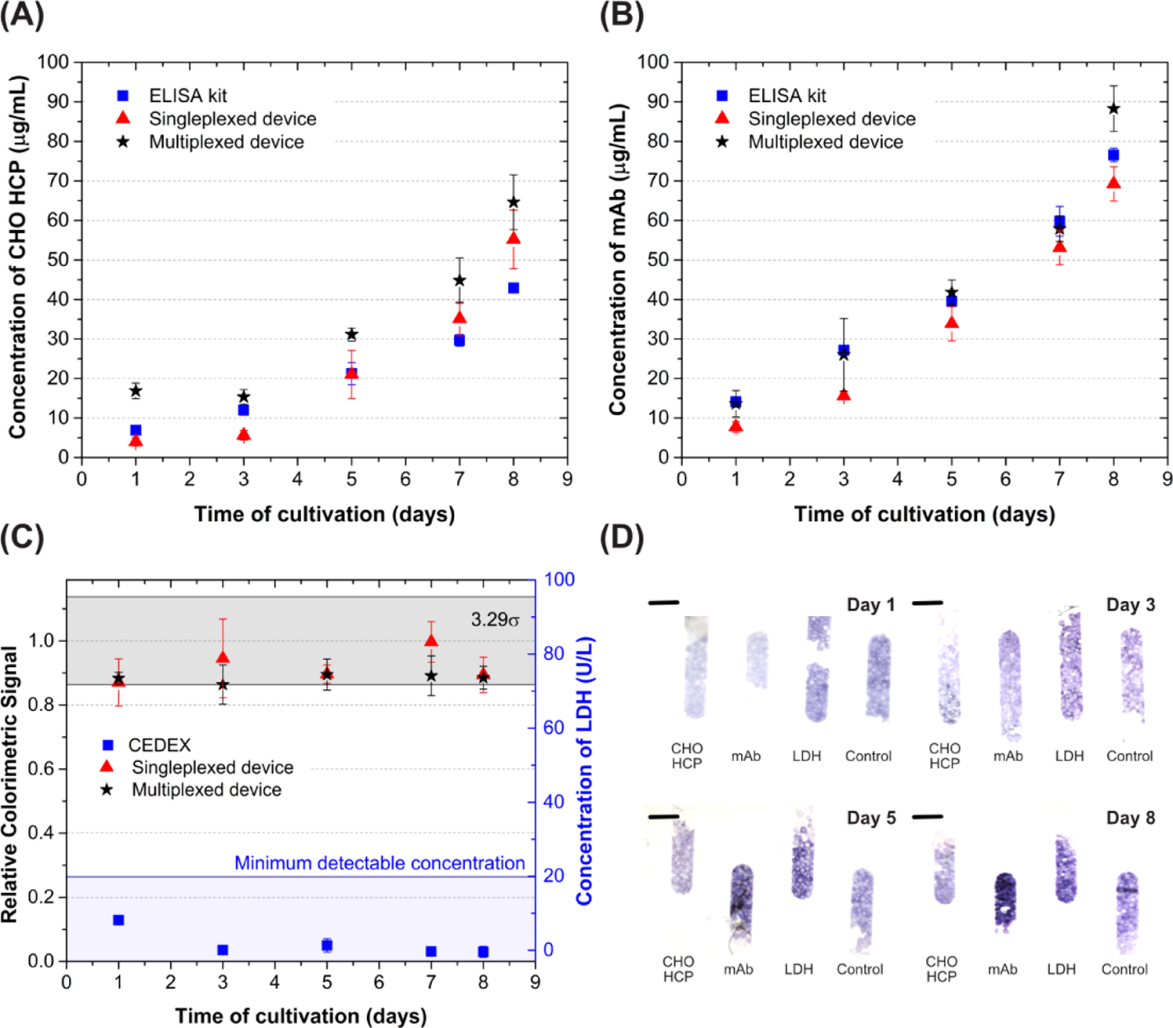
Quantification of (A) CHO HCP, (B) Rituximab, and (C) LDH in a CHO cell bioreactor over the course of 8 days. (D) Flatbed scanner images of the multiplexed measurements of bioreactor samples collected on days 1, 3, 5, and 8. For quantification using the ELISA kits, the samples were diluted 500–2000-fold. For the single-plexed device, CHO HCP and Rituximab measurements were performed diluting the samples 500- and 2000-fold, respectively. For the multiplexed measurements, all targets were measured with a 2000-fold dilution. All dilutions were performed in Cygnus dilution buffer. The concentration of CHO HCP and Rituximab were obtained using calibration curves measured with the purified molecules in the single-plexed device. In (C), relative signals were normalized to a blank measurement of Cygnus dilution buffer (value of 1.0, [LDH] = 0 ng/mL). All Cedex LDH measurements were below the detection limit of the kit.^29^ In (D), the uneven packing is caused by bead polydispersity (45–165 μm; channel height, ∼100 μm); however, this has not significantly affected the assay quantification, since the colorimetric signal was measured as the average signal of the area with beads in each column. The error bars correspond to the standard deviation of two (ELISA kit) or three independent measurements of each bioreactor sample. Scale bar = 500 μm.

Finally, the performance of both single-plexed and multiplexed devices was tested for the monitoring of a Rituximab-producing CHO cell bioreactor. A total of five samples were collected over the course of 8 days, each quantified in parallel using standard ELISA kits (CHO HCP and IgG) or the Roche Cedex platform (LDH). The measurements are shown in Figure 5, and a good agreement was observed between the standard methods, single-plexed and multiplexed devices. The multiplexed quantification of the target analytes was performed using a single dilution factor of 2000-fold throughout the entire bioreactor run. While in this particular case such conditions were suitable, it is reasonable to conceive that a feedback loop readjusting the dilution ratio would be generally required upon measuring a signal outside of the calibrated range.

To compare the methods and evaluate recovery and precision, samples collected between days 5 and 8 were considered, in which the ELISA-determined concentration of CHO HCP was above the LoD (11 ng/mL) reported in the previous section for the multiplexed device. For this device, average recoveries, relative to the ELISA measurements, of (149.5 ± 2.43) and (99.3 ± 15.0)% were obtained for CHO HCP and mAb, respectively. On the other hand, using the single-plexed device, recoveries of (115.5 ± 15.02) and (88.3 ± 2.4)% were obtained for CHO HCP and mAb, respectively. For the single-plexed CHO HCP assay and both single-plexed and multiplexed IgG assays, the performance is comparable to the spike recovery achieved using the commercial microfluidic platforms Ella (Bio-Techne) and Gyrolab for CHO HCP quantification, reported to be within the 80–120% range.^16,17^ The coefficients of variation (CVs) for all measurements of CHO HCP, IgG, and LDH (*n* = 3) were also within the 20% acceptance criteria reported for both these methods.^16,17^

The CHO HCP measurements in the multiplexed device showed an overestimation of the concentration, which can be justified by the increased signal background originated by the higher concentration of anti-IgG HRP required for the simultaneous mAb quantification assay (*i.e*., 500 ng/mL instead of 7.8 ng/mL). In this case, a calibration in the presence of both anti-IgG HRP and anti-LDH HRP antibodies is required to further improve the accuracy of the assay.

## Conclusions

A microfluidic protein analysis cartridge for the simultaneous detection of three proteins directly from bioreactor samples was developed, allowing an analysis time ranging from ∼40 min to ∼2.5 h, depending on the sensitivity requirements. As a proof of concept, the quantification of CHO HCP as key impurities, IgG as the target product, and LDH as a marker of cell viability was achieved, with limits of detection in the low ng/mL range, negligible matrix interference, and without undesired cross-reactivity between assays. The device was also demonstrated for the monitoring of a Rituximab-producing CHO cell bioreactor run, providing comparable performance to commercial ELISA kits.

Overall, considering the low reagent requirements to prepare the bead-packed device and the minimal handling times post-packing, the device showed significant potential as a versatile, scalable, and disposable protein quantification cartridge, requiring only the automation-amenable sequential flow of the target sample, spiked with corresponding detector antibodies, and enzyme substrate. In addition, these assays can potentially be extended to the detection of any protein based on affinity, characterization of mAb function *via* binding performance to its respective target,^30^ as well as applications for other therapeutic glycoproteins or growth factors in any cell culture. Furthermore, the signal transduction based on a blotting TMB substrate can be easily integrated with any imaging device without the need of monochromator or light filtering apparatuses or even visually interpreted relative to a standard sample or internal control. In collaboration with industrial partners, ongoing efforts aim at changing the device material to a scalable thermoplastic, integrating liquid handling using automated robotic stations, and achieving signal transduction *via* miniaturized optical sensors in a standalone platform to improve handling, scalability, and assay robustness.

## Supporting Information Available

Fabrication procedure, bead packing, depletion studies, assay testing with complex matrices, and concentration curves for monoclonal antibodies (PDF)
