# Supplementary material for: Multiplexed Microfluidic Cartridge for At-Line Protein Monitoring in Mammalian Cell Culture Processes for Biopharmaceutical Production
Source: ACS Sens. 2021 Mar 16;6(3):842–51. doi: 10.1021/acssensors.0c01884 (PMC8034812; doi:10.1021/acssensors.0c01884)
Supplement: Supplementary file 1 — se0c01884_si_001.pdf [file se0c01884_si_001.pdf]

Supporting Information

Multiplexed Microfluidic Cartridge for At-line Protein Monitoring in Mammalian Cell Culture Processes for Biopharmaceutical Production

Inês F. Pinto [a], Ruben R. G. Soares [a], Meeri E.-L. Mäkinen [b,c], Veronique Chotteau [b,c] and Aman Russom [a,d]*

[a] KTH Royal Institute of Technology, Division of Nanobiotechnology, Department of Protein Science, Science for Life Laboratory, 171 21 Solna, Sweden

[b] KTH Royal Institute of Technology, Department of Industrial Biotechnology, School of Engineering Sciences in Chemistry, Biotechnology and Health, 106 91 Stockholm, Sweden

[c] AdBIOPRO, Competence Centre for Advanced BioProduction by Continuous Processing, KTH, 100 44 Stockholm, Sweden

[d] AIMES, Center for the Advancement of Integrated Medical and Engineering Sciences at Karolinska Institutet and KTH Royal Institute of Technology, 100 44 Stockholm, Sweden

* Contact author:
aman@kth.se (Aman Russom)

Table of Contents

Figure S1. Fabrication of microfluidic devices………………………………………… **3**

Table S1. Bead packing in the multiplexed device…………………………..……… **4**

Figure S2. Evaluation of depletion effects in the multiplexed device…………………. **5**

Figure S3. Assay testing with complex matrices………………………………………. **6**

Figure S4. Concentration curves for monoclonal antibodies ………………………….. **7**

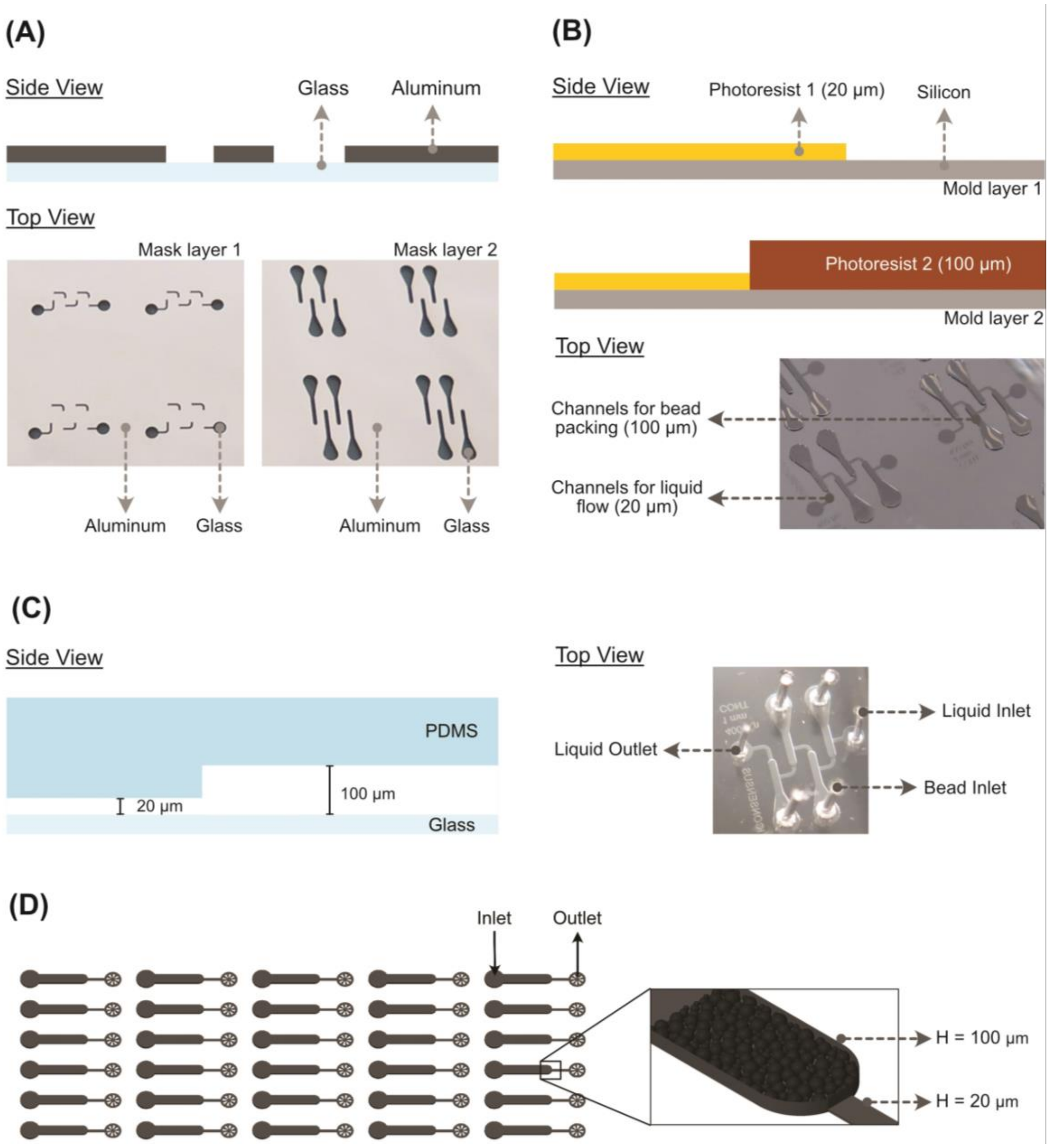

Figure S1. Fabrication of the microfluidic devices, which were designed to comprise two layers at different heights to ensure bead trapping. **(A)** Two aluminum masks, corresponding to bottom and top layer, were fabricated using direct write lithography. **(B)** The SU-8 mold was fabricated by first defining the bottom layer (20 µm) followed by the top layer (100 µm). **(C)** PDMS devices were obtained by soft-lithography replica molding and sealed against a glass slide. **(D)** An array of individual straight microcolumns was fabricated using the same techniques and used for assay optimization and singleplexed measurements.

Table S1. Sequence of steps for bead packing in the multiplexed device. Pre-conjugated beads were manually injected in the corresponding column using a pipette tip and the column inlet was subsequently closed using a 20-gauge metal plug. Loading of all solutions, including sample, was performed using a syringe pump connected to the liquid inlet of the device (positive pressure). The liquid outlet was connected to a waste reservoir. I – Injection, O – Open, X – Closed.

**Action**	**Liquid Inlet**	**Column 1**	**Column 2**	**Column 3**	**Column 4**	**Liquid Outlet**
Load Column 1	X	I	O	O	O	O
Load Column 2	X	X	I	O	O	O
Load Column 3	X	X	X	I	O	O
Load Column 4	X	X	X	X	I	O
Load all solutions	I	X	X	X	X	O

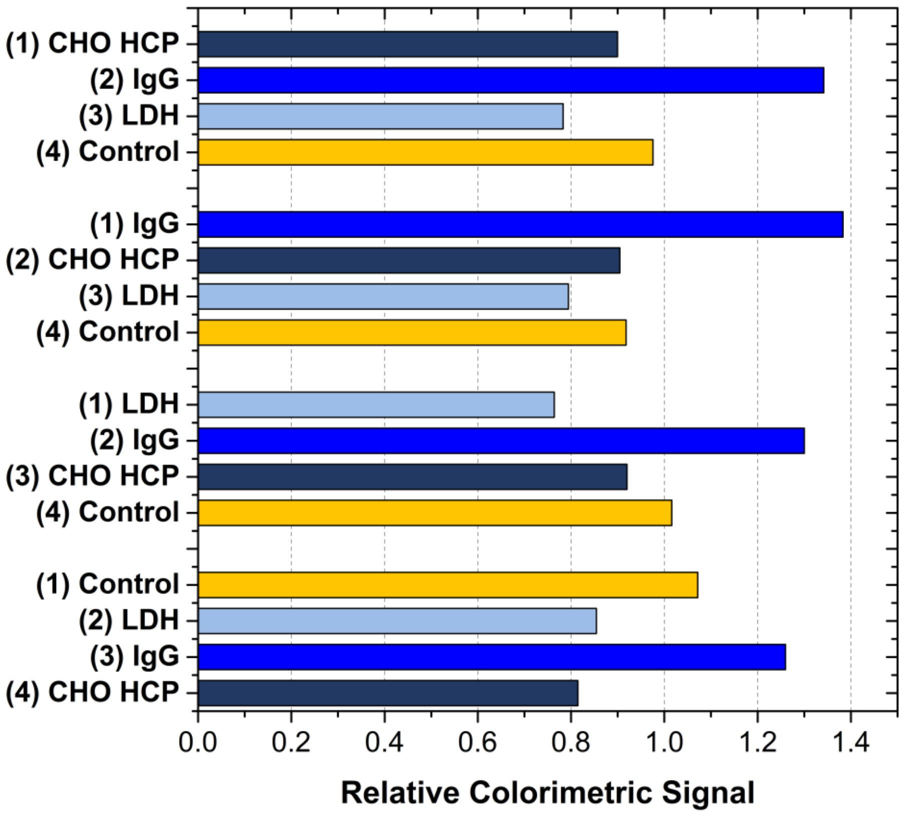

Figure S2. Evaluation of depletion effects in the multiplexed device. The beads were packed in different sequences to evaluate potential impacts on each assay in terms of analyte capture and/or signal generation. The sequence of packing is indicated from inlet (1) to outlet (4). Colorimetric signals were normalized to the average signal of all bead columns comprising each sequence, to allow a better comparison of different sequences. Concentration of the targets in the artificial mixture – [CHO HCP] = 100 ng/mL, [IgG] = 100 ng/mL, [LDH] = 30 ng/mL.

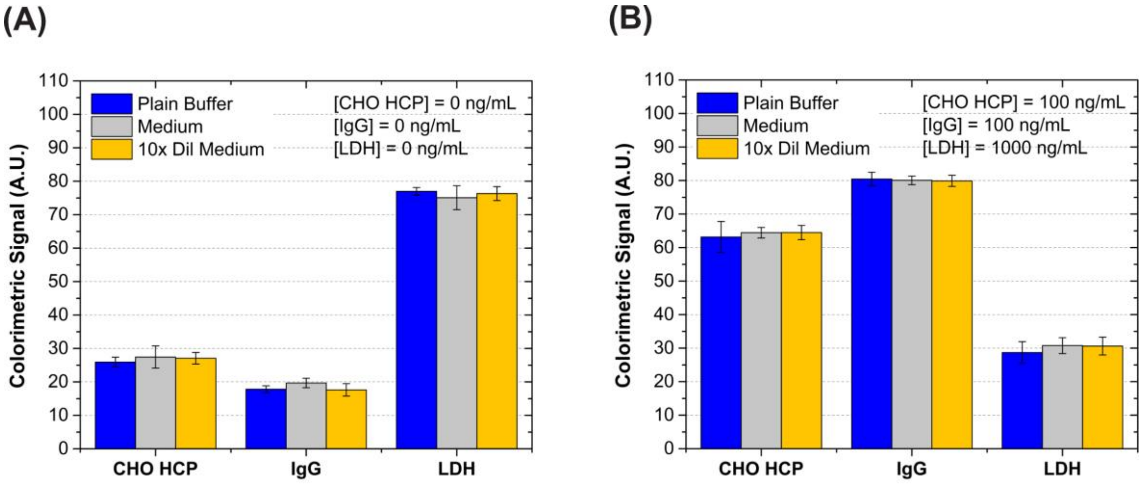

Figure S3. Evaluation of the assays using complex matrices. Samples containing a concentration of **(A)** 0 ng/mL of all target proteins and **(B)** [CHO HCP] = 100 ng/mL, [IgG] = 100 ng/mL, [LDH] = 1000 ng/mL were evaluated in plain buffer and in undiluted and 10x diluted CHO cell culture medium (FMX-8). Error bars correspond to the standard deviation of three independent measurements.

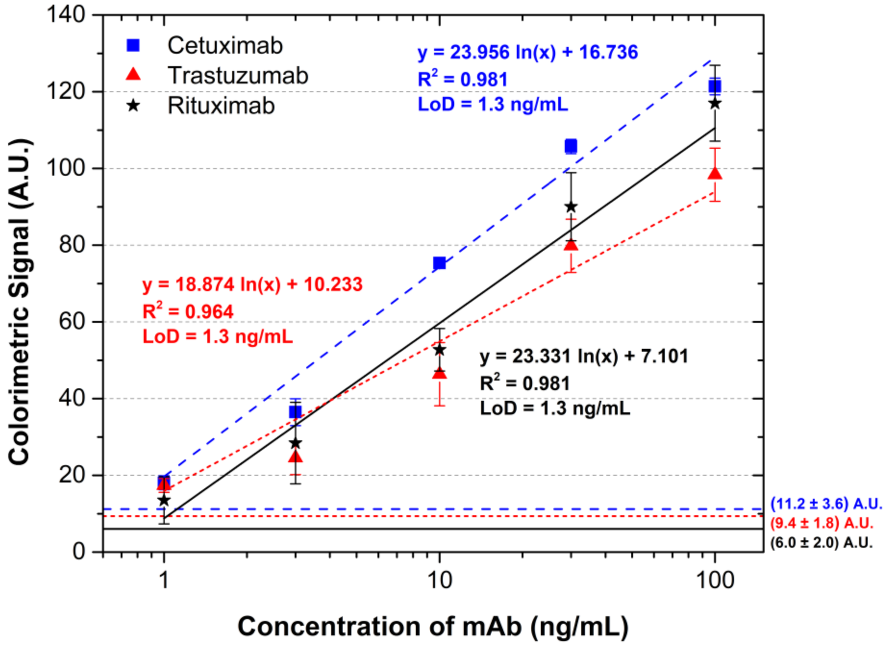

Figure S4. Concentration curves obtained for different monoclonal antibodies (mAb) using the bead-based microfluidic immunoassay. The concentration of biotinylated anti-IgG and anti-IgG HRP was 5 µg/mL and 500 ng/mL, respectively. Error bars correspond to the standard deviation of three independent measurements of each mAb concentration. Horizontal lines indicate the signal of the negative control (0 ng/mL of target mAb). Limits of detection were determined considering 3.29σ of the negative control.
